# Report of three azole-resistant *Aspergillus fumigatus* cases with TR34/L98H mutation in hematological patients in Barcelona, Spain

**DOI:** 10.1007/s15010-024-02236-7

**Published:** 2024-05-27

**Authors:** Patricia Monzo-Gallo, Ana Alastruey-Izquierdo, Mariana Chumbita, Tommaso Francesco Aiello, Antonio Gallardo-Pizarro, Olivier Peyrony, Christian Teijon-Lumbreras, Laura Alcazar-Fuoli, Mateu Espasa, Alex Soriano, Francesc Marco, Carolina Garcia-Vidal

**Affiliations:** 1 Department of Infectious Diseases, Hospital Clinic of Barcelona-IDIBAPS, University of Barcelona, Carrer de Villarroel 170, 08036 Barcelona, Spain; 2 ISCIII, Instituto de Salud Carlos III, Madrid, Spain; 3 Emergency Department, Hôpital Saint Louis, Assistance Publique-Hôpitaux de Paris, Paris, France; 4 Department of Microbiology, Hospital Clinic of Barcelona, Barcelona, Spain; 5 CIBERINFECT, Centro de Investigación Biomédica en Red, Instituto de Salud Carlos III, Madrid, Spain; 6 Facultat de Medicina i Ciències de la Salut, Universitat de Barcelona (UB), c. Casanova, 143, 08036 Barcelona, Spain

**Keywords:** TR34/L98H mutation, Invasive aspergillosis, Hematologic patients, *Aspergillus fumigatus* sensu stricto, Azole-resistant *Aspergillus*

## Abstract

**Objectives:**

We aimed to report the emergence of azole-resistant invasive aspergillosis in hematologic patients admitted to a tertiary hospital in Spain during the last 4 months.

**Methods:**

Prospective, descriptive study was performed to describe and follow all consecutive proven and probable invasive aspergillosis resistant to azoles from hematological cohort during the last 4 months. All patients had fungal cultures and antifungal susceptibility or real-time PCR detection for *Aspergillus* species and real-time PCR detection for azole-resistant mutation.

**Results:**

Four cases of invasive aspergillosis were diagnosed in 4 months. Three of them had azole-resistant aspergillosis. Microbiological diagnosis was achieved in three cases by means of fungal culture isolation and subsequent antifungal susceptibility whereas one case was diagnosed by PCR-based aspergillus and azole resistance detection. All the azole-resistant aspergillosis presented TR34/L98H mutation. Patients with azole-resistant aspergillosis had different hematologic diseases: multiple myeloma, lymphoblastic acute leukemia, and angioimmunoblastic T lymphoma. Regarding risk factors, one had prolonged neutropenia, two had corticosteroids, and two had viral co-infection. Two of the patients developed aspergillosis under treatment with azoles.

**Conclusion:**

We have observed a heightened risk of azole-resistant aspergillosis caused by *A. fumigatus* harboring the TR_34_/L98H mutation in patients with hematologic malignancies. The emergence of azole-resistant aspergillosis raises concerns for the community, highlighting the urgent need for increased surveillance and the importance of susceptibility testing and new drugs development.

## Introduction

The pathogen *Aspergillus fumigatus* has been included as critical in the WHO fungal priority pathogen list. This inclusion stems from its prevalence in human infections and the diverse challenges it poses in contemporary management. Of particular concern is the emergence of azole-resistant *Aspergillus fumigatus* strains [1]. Several countries, especially the Netherlands and Denmark, have reported increasing rates of *Aspergillus fumigatus* strains. This pattern indicates a problem of significant magnitude [2, 3]. However, in Spain, the isolations of *A. fumigatus *sensu stricto azole-resistant strains were anecdotical and mostly came from samples that were not clinically significant.

In our tertiary hospital, a national reference center, no previous cases of significant infection caused by azole-resistant *Aspergillus fumigatus* with TR34/L98H mutation in hematologic patients had been described. The clinical implications of azole resistance are of the utmost importance. Azole-resistant aspergillosis have been associated with a worse prognosis [4]. Besides, with this increase, prophylaxis strategies could be compromised.

In this article, we shed light on the concerning detection of the rapid emergence of the first three cases of azole-resistant *Aspergillus fumigatus *sensu stricto with TR_34_/L98H mutation that caused invasive aspergillosis (IA) in patients with hematologic malignancies in Spain. 

## Methods

We performed a retrospective, observational study to describe all consecutive aspergillosis diagnosed in patients with hematologic malignancies at Hospital Clinic of Barcelona, Spain. The 700-bed university center is a reference institution for a high number of hematologic malignancies and allogenic transplantations. The study included all hematologic adult patients who received an aspergillosis diagnosis between June 2023 and September 2023.

A multidisciplinary group consisting of the same hematologists, infectious disease specialists, and microbiologists has been collaboratively working for over a decade to diagnose and treat hematologic patients with infections.

The diagnosis of IA was established following the criteria published by the European Organization for Research and Treatment of Cancer (EORTC) [5].

The microbiological diagnosis for mold infections followed in our center has not changed recently. Respiratory samples were cultured in Sabouraud gentamicin chloramphenicol agar and potato dextrose agar. Fungal isolates were identified based on their colony and microscopy characteristics, and by MALDI-TOF MS. In vitro antifungal activity was studied by employing a commercial microdilution method (Sensititrer™ YeastOne ITAMYUCC panel, TREK Diagnostic Systems Ltd).

In this study, *Aspergillus fumigatus* was isolated on respiratory samples in two cases. Both isolates were sent to a reference center. *Aspergillus fumigatus *sensu stricto was ascertained by molecular identification and antifungal susceptibility was confirmed by EUCAST reference methods 9.4; available breakpoints were used to define resistance. Moreover, the presence of TR_34_/L98H mutations was determined using a real-time PCR (RT-PCR) and confirmed by DNA extraction and amplification of the *cyp*51A gene and its promoter as previously described [6]. The third case was detected using a commercialized PCR (AsperGenius^R^ 2.0 Species and Resistance TR Multiplex RT-PCR, PathoNostics^R^) in a bronchoalveolar lavage (BAL) sample which was positive for *A. fumigatus* and TR_34_/L98H mutation.

## Results and case reports

Four patients with hematologic malignancies were diagnosed with IA by *Aspergillus fumigatus* based on positive cultures and RT-PCR performed in our center between June 2023 and September 2023. One patient had azole-susceptible *A. fumigatus* isolate. And three patients presented azole-resistant *A. fumigatus* isolates or positive detection of specific azole resistance mutation by RT-PCR. Table 1 summarizes the most important characteristics of these patients. None of the patients had traveled outside of Spain in recent years. Additional information is provided in case reports.

**Table 1:** Clinical characteristics of patients with azole-resistant IA

Case	Sex, Age	Underlying diseases	Chemotherapy	Other risk factors for IA	Antifungal prophylaxis	IA diagnosis	IA syndrome	IA treatment	30-day outcome
1	M, 60	Multiple myeloma	Teclistamab (2020–2022) Daratumumab, Pomalidomide, DXM (02/2019) Carfilzomib, Lenalidomide, DXM (02/2017) VTD (05/2013)	Transplantation (autologous) Corticosteroids Viral co-infection (*influenza A*)	No	BAS culture Serum galactomannan Necropsy cultures TCTS images Clinical findings	Disseminated IA: brain, lung, heart, bowel Mucor coinfection	Liposomal amphotericin B intravenous and nebulized	Death
2	F, 57	Angioimmunoblastic T lymphoma	CHOP (05/2022) GDP (07/2022) TBF (09/2022)	Transplantation (allogenic) Mycophenolate Possible GVHD Corticosteroids Viral co-infection (*parainfluenza 3*)	Posaconazole (5 months)	Sputum culture TCTS images Clinical findings	Invasive bronchiolitis infection (small airway infection)	Liposomal amphotericin B nebulized followed by liposomal amphotericin B intravenous plus nebulized	Stable
3	F,47	Lymphoblastic B leukemia	Fludarabine Cytarabine Idarubicin (08/2023)	Neutropenia	Isavuconazole (4 weeks)	BAS galactomannan Real-time aspergillus PCR TCTS images Clinical findings	Solid pulmonary nodules with halo sign	Liposomal amphotericin B intravenous and nebulized and Anidulafungin	Improving

*IA* invasive aspergillosis, *M* male, *F* female, *DXM* dexamethasone, *VTD* bortezomib, thalidomide and dexamethasone, *CHOP* cyclophosphamide, doxorubicin, vincristine, and prednisone, *GDP* gemcitabine, dexamethasone, and cisplatin, *TBF* Tiothepa, fludarabine and busulfan, *GVHD* graft-versus-host disease, *TCTS* thoracic computed tomography scanning

### Case 1

**Fig. 1:**
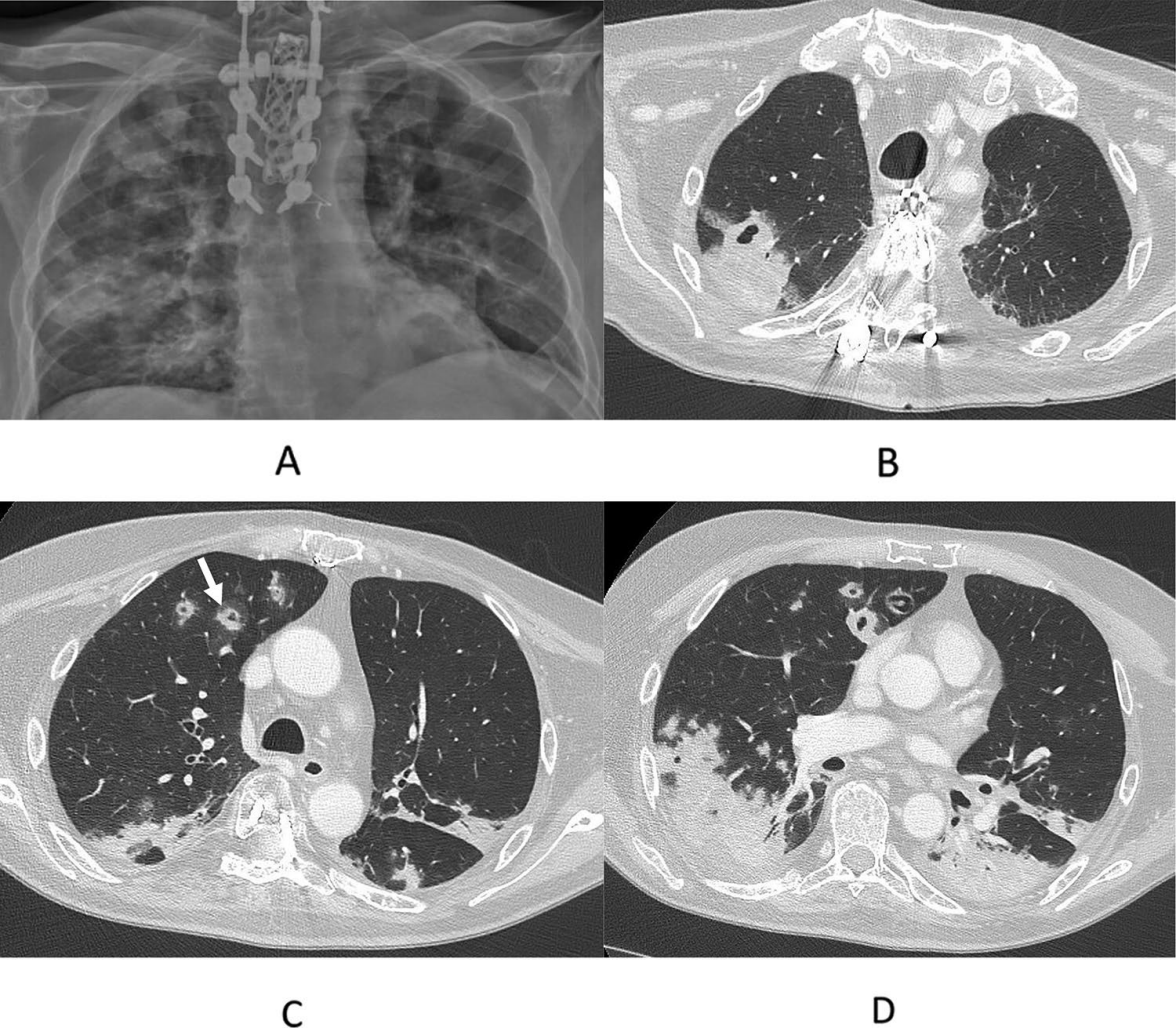
Radiological images of case 1. **A** Chest radiography. Bilateral nodular consolidations. **B**–**D** CT chest scan. Cavitated nodules and mass. Halo sign (white arrow)

A 60-year-old man was presented with severely immunosuppressed multiple myeloma with multiple lines of treatment (Table 1). The patient was receiving dexamethasone at the time of admission and trimethoprim/sulfamethoxazole as prophylaxis. He explained a few days of fever and progressive dyspnea. Chest radiography showed bilateral nodular infiltrates (Fig. 1A). A CT chest scan was performed, showing multiple nodular bilateral images (Fig. 1B–D). Serum galactomannan was positive (index 9.3) as well as influenza A PCR. The patient received isavuconazole initially but 2 days later developed a worsening of the respiratory function and was switched to nebulized and intravenous liposomal amphotericin B (l-amphotericin B). A bronchoaspirate sample was performed, isolating azole-resistant *A. fumigatus* (MICs: itraconazole > 8, voriconazole 8, posaconazole 1, and isavuconazole > 8)*.* The patient had a poor evolution with rapid neurological decline. Cranial CT scan revealed nodular images, suggesting dissemination of infection. The patient died in the next 5 days. At necropsy, *Aspergillus fumigatus* and *Rhizopus arrhizus* were isolated on various tissues.

### Case 2

A 57-year-old woman presented with an angioimmunoblastic T lymphoma who received an allogenic bone marrow transplant 8 months ago. The patient had suspected bronchiolitis obliterans by possible graft-versus-host disease (GVHD) 6 months ago and began receiving prednisone 30 mg per day. While on posaconazole prophylaxis, the patient developed respiratory symptoms consisting of cough and purulent expectoration. CT chest scan showed images consistent with bronchiolitis and bilateral nodules. A sputum sample was collected, isolating azole-resistant *A. fumigatus* (MICs: itraconazole > 8, voriconazole 8, posaconazole 1, and isavuconazole > 8). In addition, viral PCR was positive for *parainfluenza 3*. The patient received treatment with nebulized and intravenous L-amphotericin B. She is currently in follow-up.

### Case 3

A 47-year-old woman presented with a debut of lymphoblastic B leukemia who received standard induction chemotherapy with fludarabine, high-dose cytarabine and idarubicin. She underwent prophylaxis with isavuconazole from the beginning of the induction, reaching correct serum levels (4.3 ng/ml). During the neutropenic period, patient developed fever despite antibiotics. CT chest scan showed images of solid nodules with halo sign as well as infectious bronchiolitis. Patient was switched to intravenous and nebulized L-amphotericin B and anidulafungin due to suspicion of breakthrough IFI. A bronchoscopy was performed, BAL galactomannan was positive (index 3.24), no microorganism was isolated in cultures but RT-PCR for aspergillus species detection and resistance was positive detecting *A. fumigatus* and the TR_34_/L98H mutation. She improved clinically under treatment and is currently in follow-up.

## Discussion

In this report, we present the three first cases of patients with hematologic malignancies and a significant infection caused by azole-resistant *Aspergillus fumigatus* harboring the TR_34_/L98H mutation in Spain. This finding is particularly concerning, since it represents the 75% of hematologic patients diagnosed with IA in our hospital for the last months and it demonstrates the important dissemination of this problem. The TR_34_/L98H mutation was first described in the late 1980s in the Netherlands, and its clinical significance has been notable in Northern Europe. In the US, the first reported case of *Aspergillus fumigatus* with TR_34_/L98H mutation was reported in 2016 [7].

Some studies have documented a low prevalence of azole-resistant *Aspergillus fumigatus* strains in surveillance studies in Spain [8, 9]. However, in the most recent data gathered in the country from respiratory samples, regardless of clinical significance, up to 4.7% of azole-resistant *A. fumigatus *sensu stricto cases were reported, being TR34/L98H mutation the most frequent resistance mechanism that was found in seven cities across Spain, representing 2.9% of the total samples [10]. Our report outlined a rapid change in epidemiology, revealing a concerning rise in patients with hematologic malignancies in Spain experiencing significant infections due to *Aspergillus fumigatus*, showing resistance mechanisms.

Prophylaxis against filamentous fungi may help select for infections by resistant fungi [11]. However, it is important to highlight that one of our patients had never received previous antifungal treatment, supporting the other reported idea that pathogenesis of these *Aspergillus* infections began with the inhalation of spores already harboring this resistance mechanism [12]. Understanding how this phenomenon has spread to Spain or other countries remains unclear and warrants thorough investigation.

The clinical implications of the emergence of azole-resistant aspergillosis are major. First, it warrants the need to perform antifungal susceptibility testing on clinically significant positive *A. fumigatus* cultures. Second, this finding should prompt clinicians in Spain to consider that their diagnosed patients with IA, especially those based only on biomarkers, may not be adequately covered with azoles. In this regard, new PCR techniques for detecting different mutations are being employed with promising results [13] and should be incorporated into the diagnostic arsenal. In addition, in this scenario, the development and commercialization of the new antifungals appear more crucial than ever. Lastly, surveillance studies throughout the continent will become necessary as a progressive increase of resistant strains could result in a compromise of the current prophylaxis.

In summary, we describe the heightened risk of azole-resistant aspergillosis caused by *A. fumigatus* harboring the TR_34_/L98H mutation in patients with hematologic malignancies in new areas. This finding poses a significant, credible threat. Depending on its evolution, it may lead to substantial changes in the management of patients with IA.

## Data Availability

No datasets were generated or analysed during the current study.
